# Distal humerus fractures in subjects over 65 years old: about 62 cases in Moroccan population

**DOI:** 10.11604/pamj.2024.48.75.44154

**Published:** 2024-06-27

**Authors:** Anass Abaydi, Tarik Bouziani, Amine Tbatou, Jihad Radi

**Affiliations:** 1 Department of Traumatology Orthopedic Surgery, Centre Hospitalier Universitaire Hassan II, Fez, Morocco

**Keywords:** Distal humerus fractures, low-energy trauma, osteoporosis, surgical treatment, stable fixation, early rehabilitation

## Abstract

Distal humerus fractures, defined as interruptions of bone continuity below the insertion of the anterior brachial muscle, are relatively rare, representing 1 to 2% of all fractures. These fractures display a bimodal distribution, predominantly affecting young men (12-19 years old) due to violent trauma and elderly women (>80 years old) due to low-energy trauma associated with osteoporosis. This study aims to present the clinical, radiological, and therapeutic outcomes of distal humerus fractures in subjects over 65 years. A retrospective analysis of 62 cases treated surgically with plates at CHU Hassan II in Fes, Morocco, from January 2010 to December 2023 was conducted. Patients included were over 65 years old with non-pathological distal humerus fractures. Data were collected on epidemiology, fracture characteristics, surgical treatment, complications, and functional outcomes. The average age of patients was 73 years, with equal gender distribution. Most fractures resulted from simple falls (56%) and road traffic accidents (33%). Common comorbidities included hypertension, dyslipidemia, obesity, and confirmed osteoporosis. Surgical treatment with plates, predominantly using the posterior approach, showed a high consolidation rate of 89%. Complications included infection, ulnar nerve paresthesia, stiffness, malunion, and nonunion. Functional outcomes, assessed using the Mayo Clinic Elbow Performance Score, were excellent to good in 64.5% of patients, with significant pain relief and satisfactory mobility observed in most cases. In conclusion, managing distal humerus fractures in individuals over 65 is challenging due to anatomical complexity and comorbidities. Conservative surgical treatment with dual plating, particularly parallel plating with anatomical pre-molded plates, provides superior stability and favorable functional outcomes, emphasizing the importance of stable fixation and early rehabilitation.

## Introduction

Distal humerus fractures, defined as interruptions of bone continuity below the insertion of the anterior brachial muscle, are relatively rare, representing 1 to 2% of all fractures. They are characterized by a bimodal distribution according to age and gender: they typically affect young men (12-19 years old) following violent trauma and elderly women (>80 years old) following low-energy trauma, due to the high frequency of osteoporosis at this age [1]. These fractures require a multi-faceted therapeutic approach, ranging from simple immobilization to total elbow arthroplasty. Internal osteosynthesis with a plate is generally the gold standard for achieving stable fixation that allows for early mobilization [2]. The objective of our work is to present the clinical, radiological, and therapeutic results of distal humerus fractures in subjects over 65 years old. We present the analysis of clinical and radiological results with an average follow-up of 41 months in a retrospective series of 62 cases treated surgically with a plate in the orthopedics and traumatology department of CHU Hassan II between January 2010 and December 2023, compared to recent data in the literature.

## Methods

**Study design**: this study is a retrospective analysis of 62 cases of distal humerus fractures treated surgically with plates.

**Setting:** the study was conducted in the Orthopedic Surgery and Traumatology Department (A) of CHU Hassan II in Fes, Morocco. The study period spans 14 years, from January 2010 to December 2023.

**Participants:** the study included patients over 65 years old who were treated for non-pathological distal humerus fractures. The exclusion criteria were: i) patients under 65 years old; ii) patients with fractures treated orthopedically; iii) patients with incomplete medical records.

**Data collection:** data was collected using a standardized data collection sheet to gather epidemiological, anatomical, therapeutic, and evolutionary information. The collected data included: i) age and gender distribution; ii) mechanism of trauma; iii) pathological details (side of lesion, location, fracture line); iv) treatment details (surgical approach, osteosynthesis method); v) clinical and radiological outcomes.

### Variables

**Age and gender:** age of the patients and distribution between males and females.

**Mechanism of trauma:** the cause of the fracture (e.g., fall, road traffic accident).

**Comorbidities:** associated medical conditions such as hypertension, diabetes, dyslipidemia, cardiopathy, nephropathy, osteoporosis, and obesity.

**Fracture details:** type and classification of the fracture using the AO classification system.

**Surgical approach:** type of surgical approach used (posterior, lateral, medial) and the specifics of the osteosynthesis method (type of plates and screws used).

**Outcomes:** functional and radiological outcomes including consolidation rates, complications, and functional scores (e.g., MEPS).

### Surgical procedure

**Preoperative assessment:** all patients underwent standard radiographs (AP and lateral views) and CT scans with 3D reconstruction for detailed fracture analysis.

**Surgical approach:** the majority of surgeries were performed using the posterior approach (83%), with variations including trans-olecranon, paratricipital, and trans-tricipital methods. The lateral (12%) and medial (5%) approaches were used less frequently.

**Osteosynthesis:** different combinations of plates and screws were used for internal fixation, with a preference for LECESTRE anatomical pre-molded plates and 1/3 tube plates.

**Postoperative care:** systematic antibiotic prophylaxis and pneumatic tourniquet were used. Immobilization with a brachio-antebrachial plaster splint was maintained for an average of 3 weeks, followed by gradual rehabilitation from the third week.

**Follow-up and outcome evaluation:** patients were followed up for an average of 67 months. Clinical and radiological outcomes were evaluated to assess fracture consolidation and functional results. The Mayo Clinic elbow performance score (MEPS) was used to evaluate functional outcomes based on pain, mobility, stability, and daily activity. Complications such as infections, nerve injuries, stiffness, malunion, and nonunion were documented.

**Statistical analysis:** descriptive statistics were used to summarize patient demographics, fracture types, surgical treatments, and outcomes. Comparative analysis with recent literature data was conducted to contextualize the results.

## Results

**Participants:** the average age of our patients was 73 years, with extremes ranging from 65 to 92 years. The studied population showed no gender predominance with 31 men (50%) and 31 women (50%). Ninety percent of the patients were right-handed, and 67% of the fractures involved the dominant side. Regarding physical activity, 38% of our patients had reduced activity, 27% were sedentary, and 33% maintained normal physical activity.

### Descriptive data

**Mechanism of trauma:** a simple fall was the cause of the fracture in 56% of the cases, 33% were due to a road traffic accident (RTA), and 11% had other mechanisms.

**Comorbidities:** most patients had a history of comorbidities such as hypertension (HTN), dyslipidemia, and obesity, with confirmed osteoporosis. Sixty-seven percent (67%) of the patients consulted the emergency department within the first 24 hours, while 33% consulted between 1 and 24 days after the trauma, often after resorting to traditional treatment.

### Fracture and treatment details

**Figure 1:**
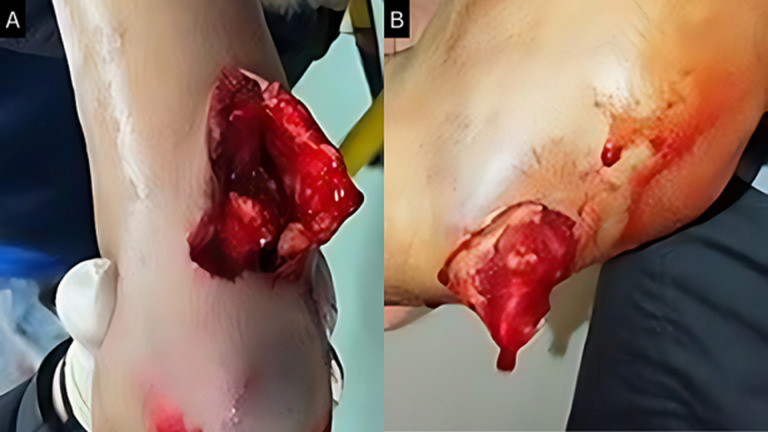
A, B) open fracture, stage 02 couchoix and duparc, of the distal humerus

**Fracture types:** at clinical examination, patients presented with a traumatic attitude of the upper limb with difficult-to-identify elbow landmarks due to edema. Skin opening was found in 10 patients (16.1%) and classified as stage 2 according to Cauchoix and Duparc classification (Figure 1). No vascular injuries were noted. Six patients had ulnar nerve paresthesias without motor involvement, and no median or radial nerve involvement. Fifty percent of the patients had associated injuries.

Standard radiographs allowed diagnosing fractures and ruling out dislocations. All patients had two elbow X-rays (AP and lateral views) and other views depending on associated injuries. The fractures were complex with at least two fragments in 38 cases (61.2%) and simple in 24 cases (38.8%), all displaced. We used the AO classification commonly used in the literature: type A (extra-articular): 21 cases (10 A2, 11 A3), type B (partial articular): 10 cases (3 B1, 7 B2); type C (intercondylar in Y or T): 31 cases (17 C1, 7 C2, 7 C3). For detailed preoperative analysis, a CT scan with 3D reconstruction was performed in 54 cases. The average time between trauma and surgical treatment was 6 days (extremes from 0 to 24 days).

**Figure 2:**
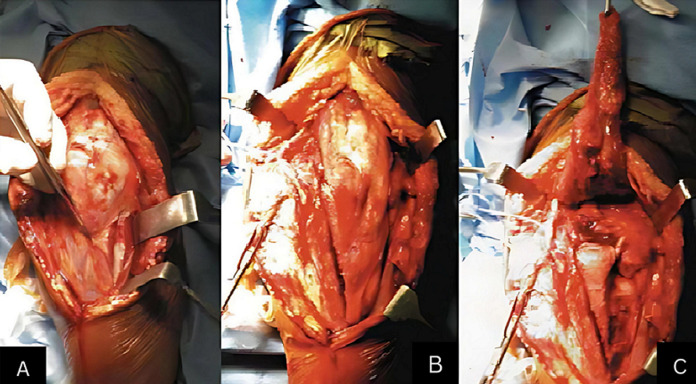
A, B, C) posterior and trans-olecranon approach of the elbow

**Figure 3:**
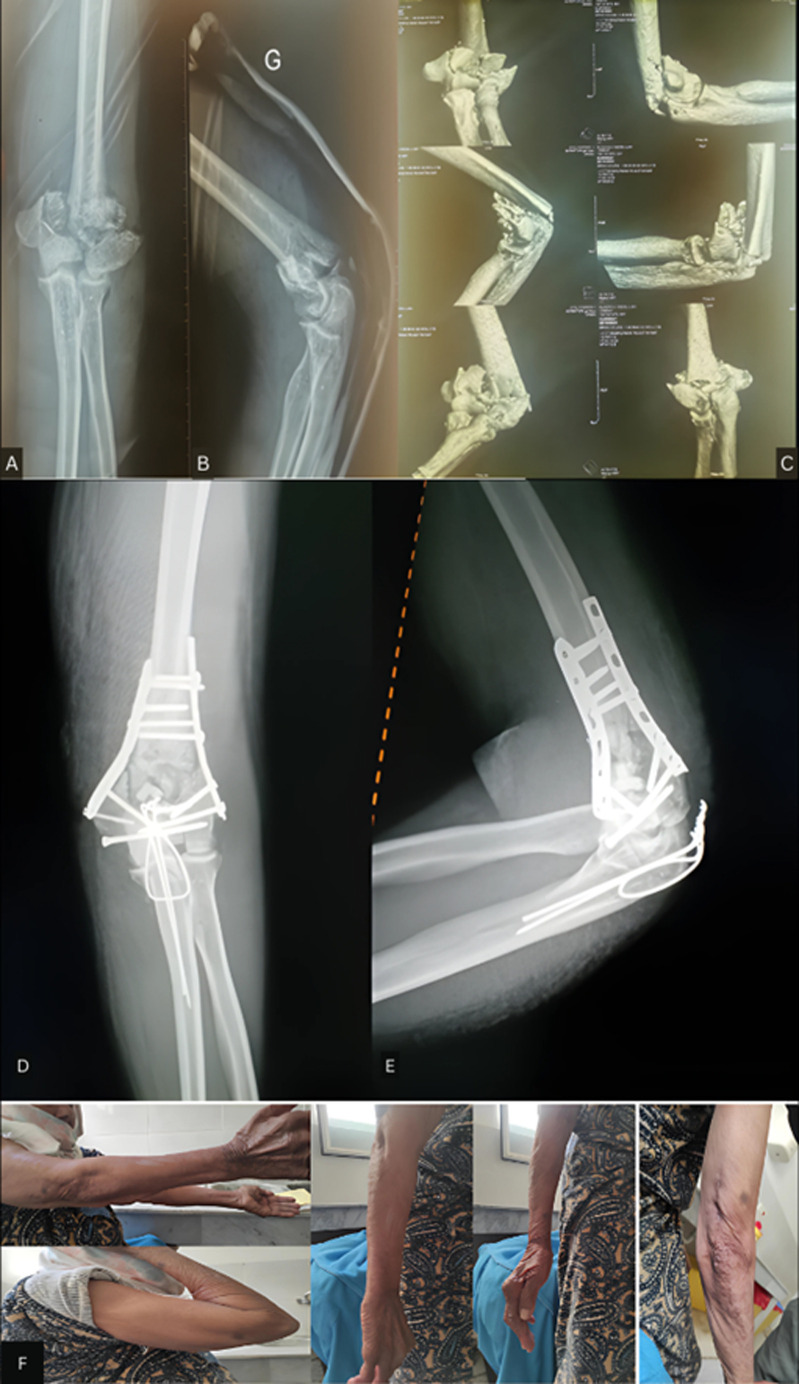
complex articular fracture; A,B) classification type C, standard elbow radiograph, anteroposterior and lateral views; C) 3D CT scan; D,E) dual plate parallel osteosynthesis with initial simplification by direct epiphyseal screwing and final bridging fixation of the olecranon osteotomy; F) highly satisfactory outcome (flexion, extension, and pronosupination) with good healing after 4 months of follow-up

**Surgical approach:** all patients underwent conservative surgical treatment with plates (LECESTRE anatomical pre-molded plates, 1/3 tube plates) with a 90° or 180° setup. General anesthesia was used for all patients. Surgery was performed in the lateral decubitus position in 83% of the cases, with the arm resting on a support and in the dorsal decubitus position in 17% of the patients. Systematic antibiotic prophylaxis and a pneumatic tourniquet at the root of the limb were used. The posterior approach was predominant (83%), including 61% trans-olecranon (Figure 2), 17% paratricipital medial and lateral, and 5% trans-tricipital. The lateral approach (12%) and medial approach (5%) were less frequent. For distal humerus osteosynthesis, 67.8% of the cases were treated with double plate (62.5% parallel setup, 37.5% perpendicular setup) (Figure 3), (Table 1, Table 2). Olecranon osteotomies were systematically osteosynthesized with pinning and cerclage. A redon drain was placed for 48 hours, antibiotic prophylaxis lasted 8 days, and brachio-antebrachial plaster splint immobilization was maintained for an average of 3 weeks.

**Table 1:** distribution according to osteosynthesis methods

Osteosynthesis material	Number of cases	Percentage
Lecestre plate alone	3	4.8%
Lecestre plate + screws	10	16.2%
Lecestre plate + 1/3 tube plate	26	41.9%
Lecestre plate + anatomical pre-molded plate	11	17.7%
Anatomical pre-molded plate + 1/3 tube plate	5	8.1%
Screws + 1/3 tube plate	7	11.3%
Only pinning	2 (waiting treatment for an open fracture followed by Lecestre plate + 1/3 plate)	
External fixation	0	0%

**Table 2:** distribution of osteosynthesis material according to anatomopathological type

Type de fracture	Type A	Type B	Type C
Lecestre plate alone	1	2	-
Lecestre plate + 1/3 tube plate	5	-	21
Lecestre plate +anatomical pre-molded plate	1	-	10
Anatomical pre-molded plate + 1/3 tube plate	2	3	
LECESTRE plate + screws	7	3	-
Screws + 1/3 tube plate	5	2	-
Only pinning	-	-	2 cas: traitement d’attente
External fixation	-	-	-

### Outcomes

**Consolidation rate and complications:** the consolidation rate was very satisfactory (89%) for distal humerus fractures and 100% for olecranon fractures. Rehabilitation began gradually from the third week. In our series, we observed 3 cases of superficial infection, 4 cases of ulnar nerve paresthesia (related to extensive neurolysis and parallel plate setup), 7 cases of stiffness (11.2%) due to prolonged immobilization and insufficient rehabilitation, 7 cases of malunion in patients with comminuted fractures, and 8 cases of nonunion due to insufficiently solid setup on osteoporotic ground. No cases of arthritis, ankylosis, or instability were noted.

**Functional outcomes:** the functional MEPS (Mayo Clinic Elbow Performance Score) was used to evaluate the results. This score includes criteria for pain (45 points), mobility (20 points), stability (10 points), and daily activity (25 points). The results were excellent (90-100 points) for 21% of the patients, good (75-89 points) for 43.5%, average (60-74 points) for 27%, and poor (<60 points) for 8%. Pain relief was obtained in 72.5% of the patients (45 cases), 10 patients had mild pain, and 7 patients had moderate pain. Forty-five patients had a mobility arc >100°, 10 patients between 50° and 100°, and 7 patients had a mobility arc less than 50°. No cases of laxity were found. 48 patients resumed their daily activity symmetrically. Radiological results show a consolidation rate of 89% >12 weeks and 55% of nonunion. The 5 cases of poor results involved open and comminuted fractures, affecting surgical reduction and early rehabilitation Table 3.

**Table 3:** results according to fracture type

Results by tracture type	Excellent	Good	Average	Poor
A2	4	6		-
A3	4	6	1	-
B1		3	-	-
B2	5	-	2	-
C1	-	10	7	-
C2	-		5	2
C3	-	2	2	3

## Discussion

Fractures of the distal humerus represent 1 to 2% of all adult fractures. The incidence of these fractures in a population of women over 60 years old is reported to be 25/100,000 per year. The distribution of these fractures is bimodal, with a peak in young men between 12 and 19 years old and a second peak in elderly women over 80 years old [3]. The epidemiological data from our series align with those in the literature. Indeed, the multicentric study conducted by the Western Orthopedic and Traumatology Society (S.O.O.) in 2007 found an average age of 80 years with a clear female predominance (80%), whereas in our study, no gender difference was observed [4]. Osteoporosis is a significant public health issue. The rate of lower humerus fractures is expected to triple by 2030 [5]. One in two women and one in five men over the age of 50 will experience an osteoporotic fracture during their lifetime. In a study on the mechanisms of osteoporotic fractures. Palvanen observed a very high rate of simple falls, reaching 97% [6]. Our own results show a slightly lower rate at 67.7%, while only 45% of the cases involved either an osteoporotic fracture or abnormal bone density. These figures highlight the significant issue posed by osteoporosis. Comparing data from different studies shows that falls remain the most frequent cause of distal humerus fractures, which aligns with our series, though there is a notable increase in fractures following road traffic accidents (RTAs). In all studied series, supracondylar and intercondylar fractures (type C) are the most common distal humerus fractures, followed by supracondylar fractures (type A). Soft tissue injuries can be caused either by the displacement of bone fragments or by direct trauma from the injuring agent. Skin lacerations affect the prognosis of these fractures by increasing the risk of infection. Most authors note a high frequency of type I injuries, including in our series.

The incidence of vascular injuries is difficult to judge in the international literature, and none were noted in our series. However, vascular examination remains systematic in elbow trauma to check for humeral artery injury. The incidence of nerve injuries is also hard to assess, with differing calculations of persistent versus transient injuries and unclear timing of deficit occurrence [1]. The average prevalence of nerve injuries in distal humerus fractures is approximately 12.3% (ranging from 0-50.9%) for transient injuries and 5.4% (0-15%) for permanent injuries. Our series shows comparable prevalences: 9.7% preoperatively and 6.4% new injuries immediately postoperatively, resulting in an overall incidence of 16.1%. The prevalence of permanent injuries is similar to that in the literature (Table 4). Distal humerus fractures represent one of the most complex situations to manage in the elderly due to the anatomical complexity of the already osteoporotic epiphysis, the proximity of nerves (radial and ulnar), and the wide variety of fracture types. Historically, conservative treatment methods like the “bag of bones” approach have been favored [7]. Methods such as external reduction followed by plaster immobilization or continuous traction have been proposed, as well as immediate rehabilitation to promote articular surface remodeling [8]. As an element of the therapeutic arsenal, conservative treatment should remain a therapeutic alternative chosen based on the patient's condition [9]. This includes non-displaced fractures, patients with unstable medical conditions, those unable to undergo surgical anesthesia, or those with advanced dementia, stroke, or paralysis [10]. Treatment consists of brief immobilization with a brachio-antebrachial-palm plaster for less than 21 days with the elbow at 60°-90° flexion, followed by gentle, early movement.

**Table 4:** distribution of distal humerus fractures according to the AO classification

Authors	Number of cases	Type A	Type B	Type C
Clavert	53	24.52%	26.41%	49%
Serrano-Maeto	68	11.76%	-	88.23%
Saragaglia	53	16%	8%	47%
Mohamed Moursy	27	22.23%	18.52%	59.25%
Our series	62	33.8%	16.2%	50%

Advances in osteosynthesis have shown promising results. Anatomically pre-molded, locked plates, reinforced Y-plates, and Lecestre plates are commonly used to ensure rigid stabilization, allowing early rehabilitation [11]. Debates persist regarding the optimal plate angle (90° vs.) [12], with recent studies favoring parallel assembly for its rigidity and resistance [13]. The use of external fixators is reserved for complex open fractures, often awaiting definitive treatment or in cases of unstable elbows post-osteosynthesis [14]. Total elbow prosthesis for these fractures was proposed by Cobb and Morrey in 1997, with satisfactory immediate results [15]. Hemiarthroplasty must be anatomical, with its indication only if the columns are preserved to ensure prosthesis stability or if they can be synthesized. The most commonly used approach is the trans-olecranon route. Observed complications include olecranon wear, olecranon pseudarthrosis, and prosthetic instability. Postoperative rehabilitation remains fundamental in managing this fracture type [16]. It aims to prevent stiffness and reintegrate the elbow into the upper limb's motor program. Consolidation typically occurs in 45 to 60 days, but this period is often extended, regardless of treatment, in cases of open or comminuted fractures. Rehabilitation should not be delayed to avoid stiffness. All authors emphasize the need for short immobilization and prolonged rehabilitation due to the slow recovery of mobility. Immediate complications such as skin laceration, vascular injuries, and nerve injuries; secondary complications such as pain, hematoma, edema, infections, secondary displacements; and late complications such as elbow stiffness, pseudarthrosis, malunion, and hardware discomfort are well-documented in the literature [1]. In 2024, François found that total elbow arthroplasties (TEA) show slight functional superiority and fewer overall complications compared to open reduction and internal fixation (ORIF), but TEA-specific complications remain a significant challenge [17]. Our MEPS (78%) is very close to ORIF studies in the literature (Table 5).

**Table 5:** comparison of the figures from our study with those from the literature

Authors	Number of cases	Age	Average MEPS	Complication rate
**Results of orif**				
Schindelar 2019	766	74	82	17%
Mohamed 2020	27	78	88	55.5%
Lopiz 2021	13	79	83	23%
Kervinen 2022	39	75.9	85	21%
Our series	62	73	78	46.8%
**Results of tea**				
Poliacomi 2016	19	74	88	35%
Barco 2017	44	71	91	52%
Strelzow 2021	40	79	90	23%
**Results of Orif Vs Tea**				
Schindelar 2019	1216 (766 vs 450)	74 vs 75	82 vs 88	17% vs 25%
Baik *et al*. 2020	71 (28 vs 43)	77.8	94 vs 81	46% vs 32%
Lopiz 2021	24 (13 vs 11)	79 vs 82	83 vs 71	23% vs 63%

MEPS: mayo clinic elbow performance score

## Conclusion

Fractures of the humeral shaft in individuals over 65 years of age are becoming increasingly common, correlating with the rise in life expectancy and the prevalence of osteoporosis. While diagnosis is now easier due to imaging innovations (such as CT with 2D and 3D reconstruction), management remains a challenge even for experienced surgeons. Achieving a stable, pain-free, and functional elbow (with a range of motion between 30°-130°) in such a population, often with multiple comorbidities and poor adherence to postoperative rehabilitation programs, is a real challenge. Orthopedic treatment is abandoned, except for certain displaced fractures, and in patients with significant surgical risk and limited functional demand. Conservative treatment with dual plating is the universal gold standard. The superiority of parallel plating over perpendicular plating, proven by numerous biomechanical and clinical studies, as well as the use of pre-contoured and locked anatomical plates, allows for greater stability of the fracture site and early initiation of rehabilitation.

### What is known about this topic

- *Distal humerus fractures are relatively rare, representing 1-2% of all fractures, and are characterized by a bimodal distribution: young men typically suffer from high-energy trauma, whereas elderly women often experience these fractures due to low-energy trauma related to osteoporosis;*
- *Internal osteosynthesis with plates is generally the gold standard for achieving stable fixation that allows early mobilization in distal humerus fractures;*
- *The incidence of these fractures is increasing in the elderly population, correlating with the rise in life expectancy and the prevalence of osteoporosis*.

### What this study adds

- *In patients over 65 years old, the study found no gender predominance in distal humerus fractures, contrary to previous studies which indicated a higher prevalence in elderly women;*
- *The use of parallel dual plating has been shown to provide better stability and functional outcomes compared to perpendicular plating, supporting the adoption of parallel plating as a standard practice in elderly patients with these fractures;*
- *Despite the complexity of distal humerus fractures in the elderly, conservative surgical treatment with dual plating has demonstrated a high consolidation rate and satisfactory functional outcomes, emphasizing its effectiveness in managing these fractures in an aging population*.
